# Autoreactive marginal zone B cells enter the follicles and interact with CD4^+ ^T cells in lupus-prone mice

**DOI:** 10.1186/1471-2172-12-7

**Published:** 2011-01-20

**Authors:** Zhenhai Zhou, Haitao Niu, Ying-Yi Zheng, Laurence Morel

**Affiliations:** 1 Department of Pathology, Immunology, and Laboratory Medicine, University of Florida, Gainesville, FL 32610, USA; 2 Department of Hematology, The First Affiliated Hospital of Sun Yat-Sen University, Guangzhou, Guandong Province, China; 3 Center For Autoimmune and Musculoskeletal Disease, The Feinstein Institute for Medical Research, 350 Community Drive, Manhasset, NY 11030, USA

## Abstract

**Backgound:**

Marginal zone B cells have been implicated in the production of autoantibodies in murine models of lupus. It has been suggested that they contribute to lupus immunopathogenesis through their enhanced effector functions and their repertoire that is biased toward autoreactive specificities. In the B6.NZM2410.*Sle.Sle2.Sle3 *(B6.TC) model of lupus, the majority of marginal zone B cells are located outside the marginal zone and inside the follicles. Genetic alterations of this strain have shown a correlation between autoimmune pathogenesis and the presence of intrafollicular marginal zone B cells. This study was designed first to strengthen our original observations and to determine how the marginal zone B cells from the lupus-prone mice respond to stimulations and interact with T cells.

**Results:**

The intrafollicular location of B6.TC MZB cells starts before disease manifestations and puts MZB cells in direct contact with CD4^+ ^T cells. Two different autoreactive B cell receptor (BCR) transgenic models showed that the expression of the *Sle *susceptibility loci enhances the presence of MZB cells inside the follicles. *In vitro*, B6.TC MZB cells were better effectors than B6 MZB cells with enhanced proliferation and antibody (Ab) production, including anti-DNA Ab, in response to stimulation with TLR ligands, immune complexes or anti-CD40. Furthermore, B6.TC MZB and CD4^+ ^T cells showed a reciprocally enhanced activation, which indicated that their contacts inside B6.TC follicles have functional consequences that suggest an amplification loop between these two cell types.

**Conclusions:**

These results showed that the NZM2410 susceptibility loci induce MZB cells to locate into the follicles, and that this breach of follicular exclusion occurs early in the development of the autoimmune pathogenesis. The enhanced responses to stimulation and increased effector functions of MZB cells from lupus-prone mice as compare to non-autoimmune MZB cells provide a mechanism by which the failure of MZB cell follicular exclusion contributes to the autoimmune process.

## Background

Systemic lupus erythematosus (SLE) is an autoimmune disease in which defects in multiple B cell subsets have long been recognized [1]. Marginal zone (MZ) B cells are enriched for autoreactive specificities through the expression of self-reactive germline-encoded BCRs [2]. MZB cells transport antigen inside the follicles [3] and are potent T-cell activators that respond more rapidly than follicular (FO) B cells to T-dependent antigen [4]. MZB cells also differentiate rapidly into plasma cells [5-9]. Finally, MZB cells respond better to T cells than FOB cells *in vitro *but not *in vivo *[10], showing that physiological barriers prevent *in vivo *activation of MZB cells [11]. These observations have led to hypothesize the existence of a tolerance checkpoint which maintains follicular exclusion of MZB cells and retains them in the MZ area where very few T cells are present. A related checkpoint that efficiently censors the entrance of autoreactive cells in the IgM^+ ^CD27^+ ^B cell compartment (the human equivalent of murine MZB cells [12,13]), has been identified [14]. The expansion of MZB cells has been directly implicated in lupus pathogenesis in some murine models [15-17], but not others [18,19]. However, their involvement through altered functions or location has not yet been assessed.

We have shown that in lupus-prone B6.TC mice that express the NZM2410-derived *Sle1*, *Sle2 *and *Sle3 *susceptibility loci [20], a large proportion of MZB cells are located inside the follicles [21]. On the other hand, NZM.TAN mice, a genetically related strain that does not produce pathogenic antibodies (Abs), present an expanded MZB cell compartment that remains in the MZ location, and expresses the negative regulator CD5, which correlates with lower activation and function [22]. Furthermore, B7-2 deficiency in B6.TC mice restores MZB cell follicular exclusion concomitant with a significant reduction in autoimmune pathology [21]. Overall, these results strongly suggested that a breach in MZB cell follicular exclusion plays a significant role in lupus pathogenesis in the B6.TC model.

In this report, we show that a large proportion of B6.TC MZB cells enter the follicles early in the disease process, before autoAb are secreted, and these intrafollicular MZB cells establish contact with CD4^+ ^T cells. We have used the anti-DNA 56R [23] and rheumatoid factor (RF) AM14 [24] heavy chain (HC) BCR transgenic (Tg) models, in which the Tg B cells are preferentially selected to the MZ compartment ([23] and Morel, unpublished). In both of these models, we showed that the expression of *Sle *susceptibility loci favors the recruitment of the Tg MZB cells to the follicles. *In vitro*, B6.TC MZB cells proliferated more and produced more IgM than B6 MZB cells in response to TLR, immune complex (IC) and CD4^+ ^T cell stimulation. Finally, B6.TC MZB cells activated CD4^+ ^T cells more than either B6 MZB cells or FOB cells. Overall, our results demonstrate that autoreactive MZB cells have a greater propensity for intrafollicular location, due at least in part to their enhanced responsiveness to a variety of stimuli. Our results also suggest that B6.TC MZB cells contribute to autoimmune pathogenesis through an enhanced mutual relationship with CD4^+ ^T cells that they encounter in the follicles.

## Results

### B6.TC MZB cells enter the follicles and interact with CD4^+ ^T cells

**Figure 1:**
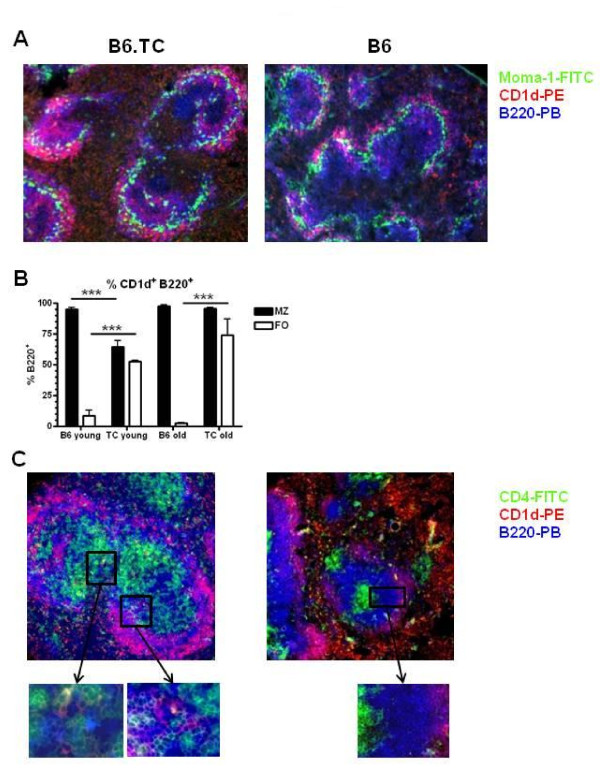
**Intrafollicular location of MZB cells in B6.TC spleens**. **A**. Representative spleen sections from 3 mo old B6.TC (left) and B6 (right) mice stained with Moma-1-FITC, CD1d-PE and B220-PB. The B220^+ ^CD1d^+ ^MZB cells show as bright pink while the other B cells show as blue. The ring of Moma-1^+ ^metallophillic macrophages delineates the MZ inner edge. **B**. Percentage of CD1d^+ ^B220^+ ^B cells relative to total B220^+ ^B cells outside (MZ) and inside (FO) the Moma-1^+ ^ring in 3 mo and 10 mo old B6 and B6.TC mice. The data show means and standard errors of the mean (SEM) calculated of 4 MZ and FO areas for each mouse. ***: p < 0.001 for t tests. **C**. Representative spleen sections from 10 mo old B6.TC (left) and B6 (right) mice stained with CD4-FITC, CD1d-PE and B220-PB. Boxed areas show multiple contacts between green B6.TC CD4 T cells and pink MZB cells, but not in the B6 spleen. Original magnification: 200X.

We have previously reported that over 80% of CD1d^hi ^B220^+ ^cells are CD21^+ ^CD23^-^, which indicates that CD1d can be used to track MZB cells by immunoflurorescence [21]. As for older mice, a large number of MZB cells were present inside the follicles of 3 mo old B6.TC mice (Figure 1A). Morphometric quantitation indicated that CD1d^hi ^B220^+ ^cells accounted for about 50% of the B cells in the follicles of 3 mo old B6.TC mice, and that this number increased to about 75% in 10 mo old mice (Figure 1B). Intrafollicular B6.TC MZB cells established numerous contacts with CD4^+ ^T cells (Figure 1C). On the contrary, the few B6 MZB cells present in the follicles were very rarely in contact with CD4^+ ^T cells (Figure 1C). CD4-MZB contacts clearly exist in 3 mo old B6.TC mice, although not as extensively as in older mice (data not shown). In addition to the MZB cell intrafollicular location, the scattering of CD4^+ ^T cells across the B6.TC follicles facilitates CD4-MZB contacts. Overall, these results show that B6.TC MZB cells are present inside the follicles in young mice, before autoimmune manifestations are manifested [20], and that they establish numerous contacts with CD4^+ ^T cells.

### The expression of the *Sle *susceptibility loci increases the intrafollicular location of MZB cells

**Figure 2:**
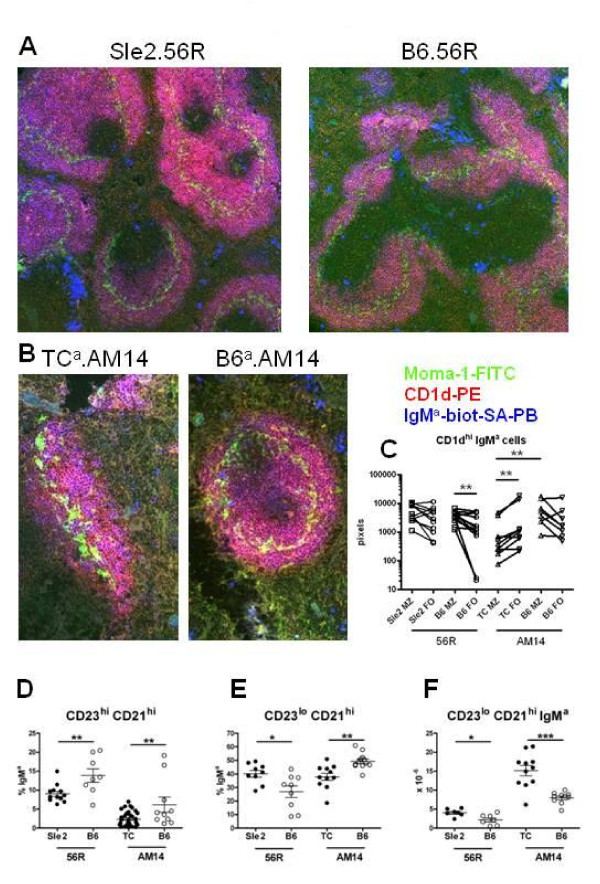
**Intrafollicular location of 56R and AM14 HC Tg MZB cells**. Representative spleen sections of B6.*Sle2*.56R and B6.56R (**A**, 100X) and B6.TC.AM14.IgH^a/b ^and B6.AM14.IgH^a/b ^**(B**, 200X**) **mice stained with Moma-1-FITC, CD1d-PE, and IgM^a^-biotin-SA-PB**. C. **Quantitation in pixels corresponding to the stain combination specific for each cell type of CD1d^+ ^IgM^a ^cells in the MZ (pink) and corresponding FO (blue) B cell areas in B6.*Sle2*.56R, B6.56R, B6.TC.AM14.IgH^a/b ^and B6.AM14.IgH^a/b ^mice. Paired MZ and FO values within a strain were compared with the Wilcoxon signed ranked test, and the B6.TC.AM14.IgH^a/b ^and B6.AM14.IgH^a/b ^MZ values were compared with the Mann-Whitney test. CD21^hi ^CD23^hi ^representing the T2 and MZB precursor cells expressed as the percentage of transgenic IgM^a ^cells **(D)**, and percentage of CD21^hi ^CD23^lo ^MZ B cells expressed as the percentage of transgenic IgM^a ^cells (**E) **and their absolute numbers (**F) **in B6.*Sle2*.56R and B6.56R and B6.TC.AM14.IgH^a/b ^and B6.AM14.IgH^a/b ^mice. Graphs show means and SEMs, and the statistical significance of Student t tests. *: p < 0.05, **: p < 0.01, ***: p < 0.001.

To further evaluate whether the autoimmune susceptibility loci carried by the B6.TC mice, we used two autoreactive B cell receptor HC Tg models that have been widely used to study B cell tolerance mechanisms, 3H9/56R and AM14 [25]. B cells carrying the DNA-specific 56R HCTg are preferentially recruited in the MZB compartment [23] and the expression of the *Sle2 *locus expands the size of the 56R MZB compartment [26]. We used immunofluorescent staining of spleen sections to determine whether this recruitment of these Tg B cells to the MZ was associated with a breach of follicular exclusion. As expected, a large number of IgM^a ^Tg B cells expressed the MZB marker CD1d in both B6.*Sle2*.56R and B6.56R spleens (Figure 2A). The percentage of CD1d^+ ^B cells inside the follicles was significantly higher in B6.56R than in B6 (34.56 ± 4.71 vs. 2.5 ± 0.65%, p = 0.002; compare Figure 2A and 2C to Figure 1A and 1B). However, CD1d^+ ^IgM^a ^cells were in significantly higher numbers in the MZ than in the FO areas in the B6.56R spleens (Figure 2C). In contrast, they were in equivalent numbers in the MZ and FO areas of the B6.*Sle2*.56R mice. This indicated that 56R MZB cells locate to the follicles with a higher frequency than non-Tg MZB cells, and that the *Sle2 *lupus susceptibility locus further increases this migration.

When paired with endogenous Vκ8, the AM14 HC Tg encodes for anti-IgG2a^a ^IgM^a ^RF [24]. In B6.TC.AM14.IgH^a/b ^and B6.AM14.IgH^a/b ^mice, the AM14 HC Tg B cells differentiate in the presence of the AM14 cognate antigen encoded by the endogenous IgG2a^a ^on either the autoimmune B6.TC or the control B6 background. In these mice, the great majority (> 90%) of IgM^a ^cells express the AM14 HC Tg, but some IgM^a+ ^cells express the endogenous IgH^a ^HC. However, there was no difference between IgH^b ^and IgH^a ^MZB cell distribution on either non-Tg B6 or B6.TC background (data not shown). This indicated that the endogenous non-transgenic B cells do not contribute to the results reported below, and that the Tg B cells accounted for the differences in MZB cell distribution between non-Tg and AM14 Tg mice. A large number of IgM^a ^cells expressed CD1d in both B6.TC.AM14.IgH^a/b ^and B6.AM14.IgH^a/b ^spleens (Figure 2B), indicating again that these HC Tg B cells were preferentially recruited to the MZB compartment. As for B6.56R, the proportion of CD1d^+ ^IgM^a ^MZB cells that breached follicular exclusion was greater in B6.AM14.IgH^a/b ^than in B6 mice (30.50 ± 7.23 vs. 2.5 ± 0.6%, p = 0.007; compare Figure 2B and 2C to Figure 1A and 1B). The *Sle1-3 *loci expressed in B6.TC mice significantly increased the shift of CD1d^+ ^IgM^a ^MZB cells toward the follicles, with a significantly higher proportion of CD1d^+ ^IgM^a ^in the follicles than in the MZ in B6.TC.AM14.IgH^a/b ^mice, but not in B6.AM14.IgH^a/b ^mice (Figure 2B and 2C). In addition, there were significantly more CD1d^+ ^IgM^a ^cells in the MZ of B6.AM14.IgH^a/b ^than B6.TC.AM14.IgH^a/b ^mice (6649 + 2169 vs. 1232 + 597 pixels, respectively; p = 0.01), and the proportion of CD1d^+ ^IgM^a ^cells with an intrafollicular location was significantly higher in B6.TC.AM14.IgH^a/b ^than in B6.AM14.IgH^a/b ^spleens (70.11 ± 4.14 vs. 30.50 ± 7.23%, p < 0.001). Overall these results show in two models that Tg B cells expressing an autoreactive HC are not only preferentially selected to the MZB compartment, but they are also more likely to breach follicular exclusion. Moreover, expression of the *Sle *susceptibility loci enhances the migration of the HC Tg MZB cells into the follicles, more strongly when the three loci are expressed than with just *Sle2*.

We examined whether the intrafollicular location of Tg B cells in the lupus-prone background correlated with the size of the MZ B cell precursor or MZB cell compartments. We have previously shown that B6.TC mice have a markedly reduced MZB cell precursor subset [22]. Here we showed that the percentage of transgenic IgM^a ^B cells in the CD23^hi ^CD21^hi ^subset, which contains the precursors of the Tg MZB cells, was reduced in both B6.*Sle2*.56R and B6.TC.AM14.IgH^a/b ^mice as compared to their B6 controls (Figure 2D). There was however no difference for the absolute number of these cells between the lupus prone and B6 background for either transgene (0.87 + 0.12 vs. 1.71 + 0.53 × 10^6 ^cells for B6.*Sle2*.56R vs. B6.56R; 1.08 + 0.25 vs. 0.93 + 0.27 × 10^6 ^cells for B6.TC.AM14.IgH^a/b ^vs. B6.AM14.IgH^a/b^). The proportion and number of Tg MZB cells was significantly higher in B6.*Sle2*.56R than in B6.56R mice (Figure 2E and 2F), as previously reported [26]. In the B6.TC.AM14.IgH^a/b ^mice, the proportion Tg MZB cells was lower, but their absolute number was significantly higher than in B6.AM14.IgH^a/b ^mice (Figure 2E and 2F). These results suggest that the selection of the B cells to the MZB subset, possibility bypassing or staying for a shorter period of time in the MZ B precursor compartment, and the size of the MZB cell compartment may be involved in their propensity for intra-follicular migration.

**Figure 3:**
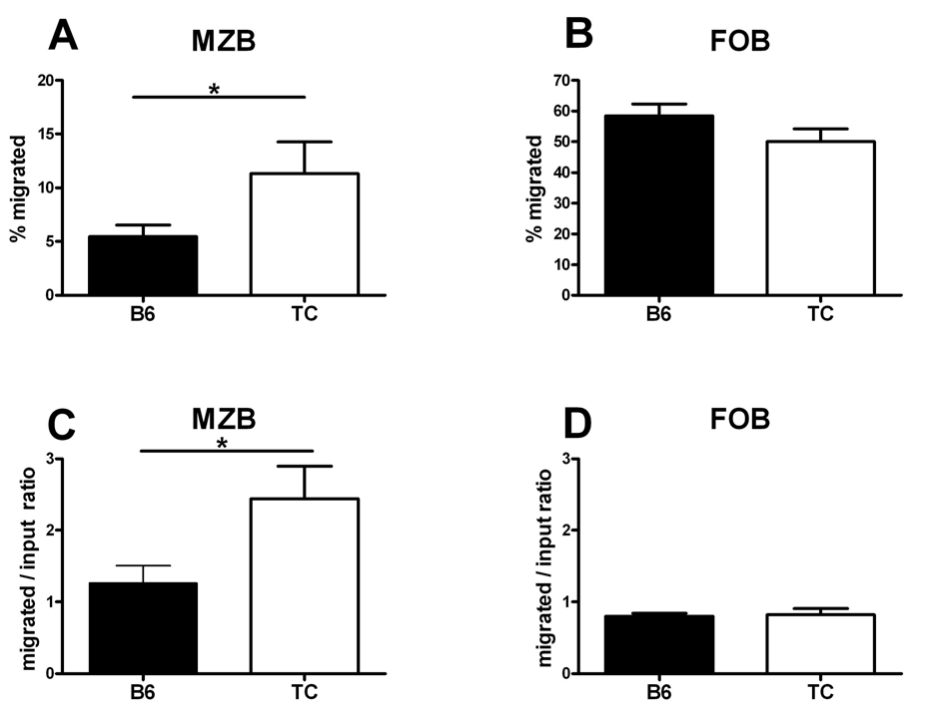
**Spontaneous migration of B6 or B6.TC B cells in transwell assays in the absence of stimulation**. Percentage of migrated cells that presented a MZB (IgM^+ ^CD23^- ^CD21^hi^) phenotype (**A**) or a FOB (IgM^+ ^CD23^+ ^CD21^int^) phenotype (**B**). **C**. Ratios of the percentage of spontaneously migrated MZB cells over the percentage of MZBs in input B cells **D**. Ratios of the percentage of spontaneously migrated FOB cells over the percentage of FOBs in input B cells. Graphs show means and SEMs, and the statistical significance of Student t tests. *: p < 0.05.

MZB cells migrate back and forth between the marginal and follicular zones transporting antigen to the follicle through a process involving CXCL13 and S1P [27]. We have previously shown that B6.TC MZB cells express higher levels of CXCR5, the CXCL13 receptor, and that they migrate more in response to CXCL13 and S1P than B6 MZB cells [21]. MZB cells from B6.TC mice have in fact a significantly enhanced propensity to migration even in the absence of any stimuli. About 1% of splenic B cells from B6 and B6.TC mice migrated through transwells in medium alone. A significantly greater percentage of these splenic migrated cells were MZBs in B6.TC than in B6 mice (Figure 3A), while no difference was observed for FOBs (Figure 3B). Since B6.TC mice have more MZB cells than B6, the compared the ratios of spontaneously migrated MZB cells to the initial MZB cell input, and obtained a significant difference between B6.TC and B6 mice (Figure 3C). On the contrary, the ratio of spontaneously migrated FOB cells over the FOB cell input was equivalent between the two strains (Figure 3D). This indicated that B6.TC MZB cells have an increased propensity for migration, which is likely to disrupt their steady state balance between the marginal and follicular zones.

### B6.TC MZB cells proliferate more than B6 MZB cells

**Figure 4:**
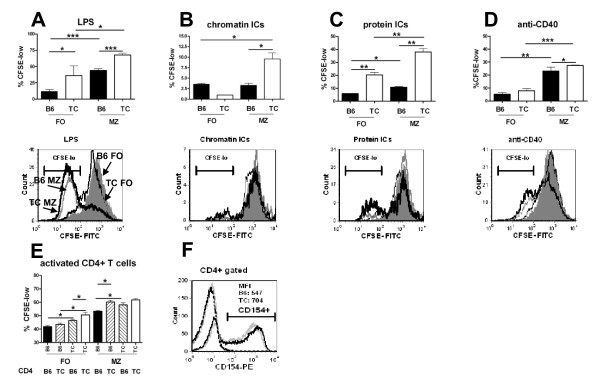
**Proliferative response of FOB or MZB cells from B6 or B6.TC mice**. Proliferation after 3 d in culture with LPS (**A**), chromatin ICs (**B**), protein ICs (**C**), anti-CD40 (**D**), or activated CD4 T cells (**E**). Proliferation was assessed by CFSE dilution as the percentage of B220^+ ^cells in the last two cell division cycles (CSFE-low). Means and SEM, Student t test comparisons: *: p < 0.05; **: p < 0.01, ***: p < 0.001. For A-D, representative histograms indicating the CFSE-low gate for B220-gated cells are shown for each stimulatory condition, with filled gray histograms corresponding to B6 FOB, thin black lines to B6.TC FOB, gray lines to B6 MZB and thick black lines to B6.TC MZB cells. **F**. Representative histogram showing surface CD154 expression on B6.TC (black) and B6 (gray) CD4^+ ^T cells without (dotted lines) or with PMA/ionomycin stimulation (solid lines). CD154 median fluorescence intensity (MFI) is indicated for activated cells gated by the horizontal markers.

We compared the proliferation of sorted B6 and B6.TC FOB and MZB cells in response to a range of stimuli in 3 d cultures. Overall, B6.TC B cells proliferated more than B6 B cells and MZB cells proliferated more than FOB cells, leading to the greatest proliferation in B6.TC MZB cells (Figure 4). More specifically, B6.TC MZB cells proliferated more than B6 MZB cells in response to LPS (Figure 4A) and CpG (data not shown). Among the four types of B cells tested, only B6.TC MZB cells proliferated to anti-chromatin ICs (Figure 4B), and B6.TC MZB cells proliferated the most to non-autoreactive protein ICs, with the lowest response observed in B6 FOB (Figure 4C). Finally, FOB cells responded poorly to anti-CD40 as compared to MZB cells, and among these, B6.TC MZB cells proliferated significantly more than B6 MZB cells to this stimulation (Figure 4D).

As for the non-cellular stimuli evaluated in Figure 4A-D, PMA and ionomycin-activated B6 CD4^+ ^T cells induced a titrated proliferative response in which B6.TC MZB cells responded more than B6 MZB cells (Figure 4E). B6.TC CD4^+ ^T cells induced a similarly high proliferation in both B6 and B6.TC MZB cells. The PMA and ionomycin stimulation induced a higher level of CD154 expression B6.TC than in B6 CD4^+ ^T cells (Figure 4F), which suggested that the difference between B6 and B6.TC MZB response could be overcome by a very strong stimulation. In support of this hypothesis, only LPS still induced a significantly higher proliferation in B6.TC than B6 MZB cells in 5 d cultures (data not shown). Overall, these results showed that B6.TC MZB cells proliferated more than B6 MZB cells in response to TLR ligands, IC or CD4^+ ^T cell stimulation.

### B6.TC MZB cells secrete more IgM in response to multiple stimuli, including CD4^+ ^T cells

**Figure 5:**
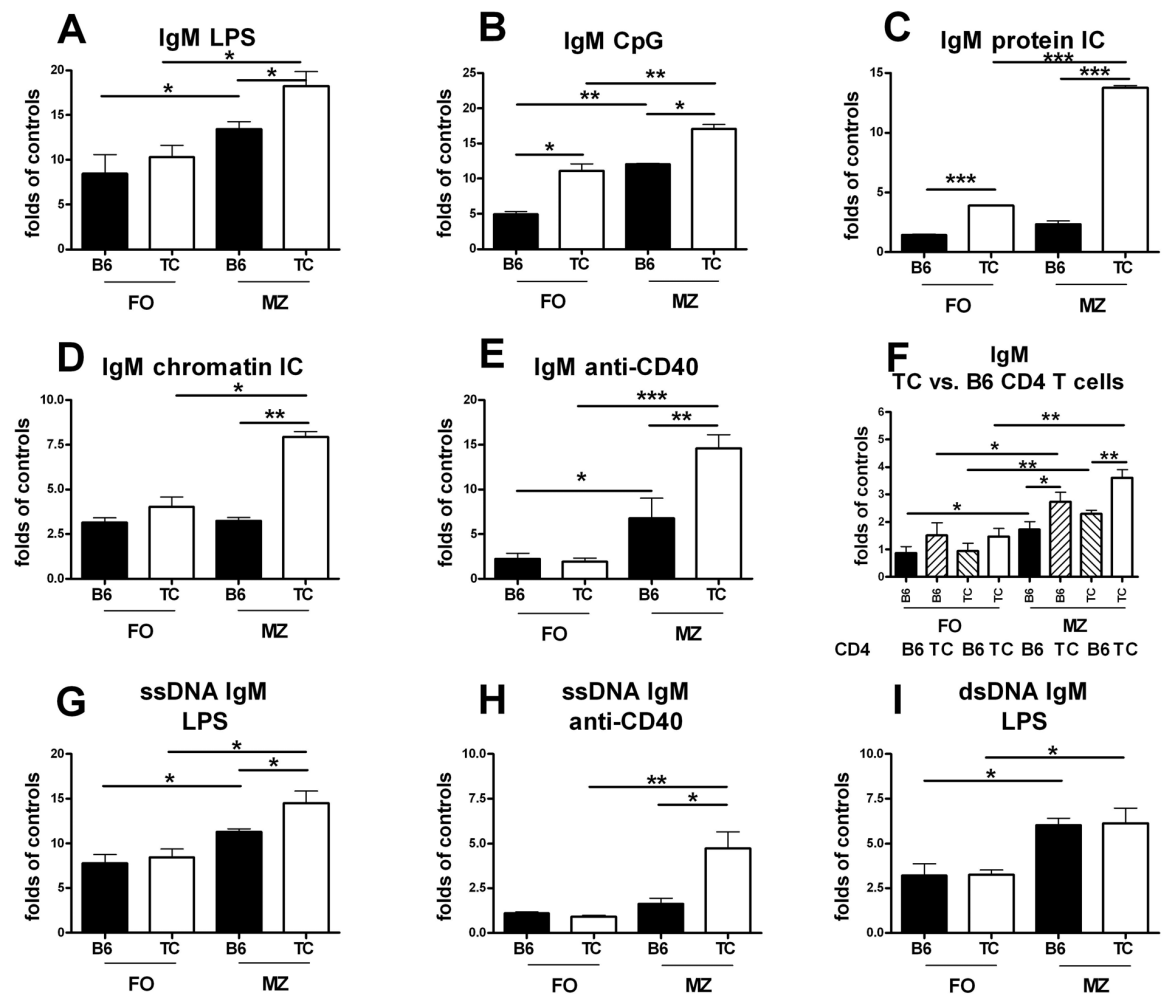
**Antibody production of FOB or MZB cells from B6 or B6.TC mice**. Total IgM (**A-F**), anti-ssDNA (**G-H**) and dsDNA IgM (**I**) production in supernatants from 5 d cultures with LPS (**A, G, I**), CpG (**B**), protein ICs (**C**), chromatin ICs (**D**), anti-CD40 (**E, H**), and B6 or B6.TC CD4^+ ^T cells (**F**). Results are expressed as fold increase over the IgM production by the corresponding B cell type and strain in the absence of any stimulation. Results are representative of two independent assays with 3 mice per strain in each. Means and SEM, Student t test comparisons: *: p < 0.05; **: p < 0.01, ***: p < 0.001.

B6.TC MZB cells, but not FOB cells, spontaneously secreted more IgM than B6 MZB cells (p < 0.01, Table 1). For this reason, we compared IgM secretion between MZB and FOB cells from both strains with absolute values (Table 1), as well as fold induction by each stimulus over the no-stimulation controls. B6.TC MZB cells secreted higher amounts of IgM than B6 MZB cells in response to LPS, CpG, chromatin or protein ICs, or anti-CD40 (Figure 5A-E and Table 1). It should be noted that B6.TC MZB cells were highly responsive to ICs as compared to the three other types of B cells (Figure 5C and 5D). As observed for proliferation (Figure 4F), the production of IgM increased as the result of the strain of origin of both B and CD4^+ ^T cells, and the type MZ or FO of B cells. This resulted in the highest increase in IgM production (over 3.5 fold on average) provided by B6.TC T cells stimulating B6.TC MZB cells (Figure 5F).

**Table 1:** Ab production from B6 and B6.TC FOB and MZB cells in response to various stimuli in 5 d cultures

	B6 FO		TC FO		B6 MZ		TC MZ	
	**mean**	**SD**	**mean**	**SD**	**mean**	**SD**	**mean**	**SD**

	total IgM							

control	0.09	0.02	0.10	0.01	0.08	0.01	0.12**	0.01
LPS	0.57	0.02	0.67	0.09	0.68	0.17	1.31***	0.06
CpG	0.26	0.03	0.59**	0.01	0.57	0.01	0.79*	0.05
protein ICs	0.08	0.00	0.21**	0.03	0.11	0.01	0.63***	0.01
chromatin ICs	0.16	0.01	0.21	0.00	0.15	0.12	0.37*	0.01
anti-CD40	0.11	0.03	0.11	0.04	0.32	0.02	0.95***	0.08
B6 CD4	0.19	0.02	0.19	0.03	0.22	0.05	0.24	0.01
TC CD4	0.31	0.05	0.30	0.02	0.35	0.03	0.37	0.04

	ssDNA IgM							

control	0.05	0.00	0.07	0.02	0.06	0.01	0.06	0.01
LPS	0.40	0.09	0.55*	0.01	0.70	0.04	0.79	0.07
anti-CD40	0.06	0.01	0.06	0.01	0.10	0.03	0.25**	0.07

	dsDNA IgM							

control	0.05	0.00	0.06	0.01	0.05	0.00	0.06	0.00
LPS	0.17	0.06	0.20	0.04	0.33	0.04	0.35	0.08

Mean ODs and standard deviations (SD) of ELISA assays performed on supernatants diluted 1:10 from one representative experiment.

* indicates the level of significance of t tests between B6 and B6.TC FOB cells on the left or between B6 and B6.TC MZB cells on the right. *: p < 0.05; **: p < 0.01, ***: p < 0.001.

Anti-DNA Abs were not produced by any type of B cells in absence of stimulation (Table 1). Both LPS and anti-CD40 induced a significantly higher production of anti-ssDNA IgM in B6.TC MZB cells than in B6 MZB cells (Figure 5G-H). Finally, anti-dsDNA IgM was produced in higher amounts in MZB cells than in FOB cells stimulated with LPS, regardless of the strain of origin (Figure 5I). Anti-CD40 induced only a small amount of anti-dsDNA IgM, and there was a trend for higher production by B6.TC MZB cells (data not shown). The other stimulatory conditions failed to induce anti-DNA IgM above control (data not shown). These data demonstrate that B6.TC MZB cells secrete higher amounts of Abs, including autoAbs, in response to TLR ligation, IC or CD4^+ ^T cell stimulation.

**Figure 6:**
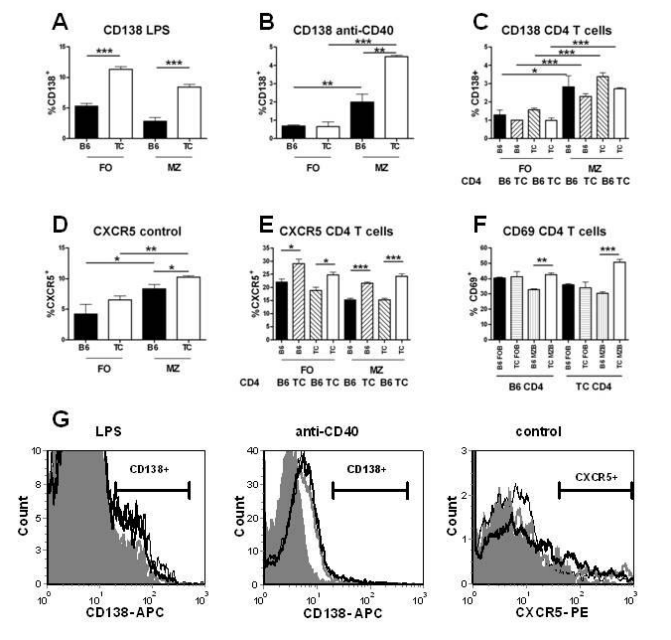
**Induction of effector cell surface markers in FOB or MZB cells from B6 or B6.TC mice**. CD138 (**A-C**), CXCR5 (**D-E**), and CD69 (**F**) induction in 5 d cultures with the indicated stimulatory conditions, including CD4^+ ^T cells from B6 or B6.TC origin. Results are representative of two independent assays. Student t test comparisons, or Dunnett's Multiple Comparison Tests when multiple groups were compared: *: p < 0.05; **: p < 0.01, ***: p < 0.001. J. **G**. Representative histograms showing CD138 and CXCR5 staining. Filled gray histograms: B6 FOB, thin black lines: B6.TC FOB, gray lines: B6 MZB and thick black lines: B6.TC MZB cells.

Both LPS-stimulated B6.TC MZB and FOB cells expressed significantly more CD138, a plasma cell marker, than the B6 B cell equivalent (Figure 6A and 6G). In contrast, only MZB cells expressed CD138 in response to anti-CD40, and the response from B6.TC MZB cells was about 2 folds higher than that of B6 MZB cells (Figure 6B and 6G). Finally, CD4^+ ^T cells induced a greater CD138 expression in MZB than in FOB cells, but there was no difference between B6 and B6.TC (Figure 6C). As for B cell proliferation, this discrepancy between stimulation with anti-CD40 and CD4^+ ^T cells maybe due to the low level of CD154 expression by CD4^+ ^T cells from young mice. CXCR5 expression is one of the determinants of MZB cell migration to the follicles [3]. As previously reported [21], CXCR5 expression was higher on B6.TC than B6 MZB cells (Figure 6D and 6G). Interestingly, B6.TC CD4^+ ^T cells induced a higher CXCR5 expression than B6 CD4^+ ^T cells in all four groups of B cells (Figure 6E), suggesting that CD4^+ ^T cells may contribute to CXCR5 expression on B6.TC MZB cells. Finally, B6.TC MZB cells responded significantly more than B6 MZB cells to either B6 or B6.TC CD4^+ ^T cells by expressing early activation marker CD69, with again the combination of B6.TC MZB and CD4^+ ^T cells resulting in the highest CD69 expression on B cells (Figure 6F).

### B6.TC MZB cells induce a greater proliferation and activation in CD4^+ ^T cells

**Figure 7:**
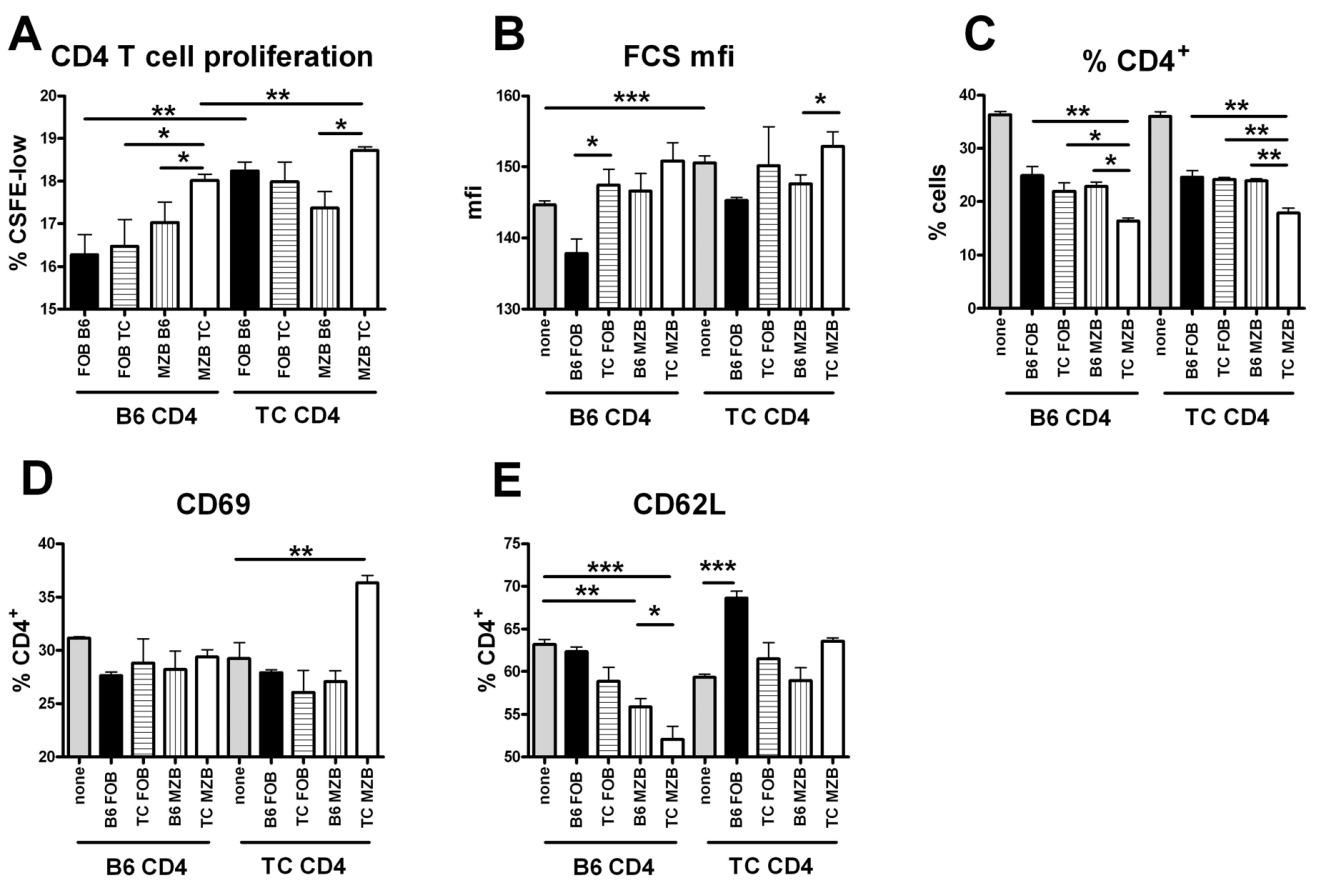
**CD4^+ ^T cell proliferation and activation in response to FOB or MZB cells from B6 or B6.TC mice**. **A**. Proliferation in 5 d cultures. **B**. Forward scatter mean fluorescence intensity (mfi). **C**. Percentage of CD4^+ ^T cells. CD69 (**D**) and CD62L (**E**) expression. All cells were gated on live CD4^+ ^T cells. Proliferation was assessed with CFSE dilution as the percentage of CD4^+ ^T cells in the last two cell division cycles (CSFE-low). Results are representative of two independent assays. Means and SEM and Dunnett's Multiple Comparison Tests. *: p < 0.05; **: p < 0.01, ***: p < 0.001.

MZB cells are better activators of CD4^+ ^T cells than FOB B cells [4]. To test whether B6.TC MZB cells were better activators of CD4^+ ^T cells than B6 MZB cells, we analyzed T cells responses in criss-cross co-cultures in which the MZB cells were either of B6 or B6.TC origin. FOB cells were used as controls. We also addressed whether the B6 or B6.TC origin of CD4^+ ^T cells impacted their response to MZB cells. We have shown that B6.TC CD4**^+ ^**T cells are more activated than B6 CD4**^+ ^**T cells [28], which was confirmed in these *in vitro *experiments (Figure 7B, D, E). B6 CD4^+ ^T cells proliferated more in response to B6.TC MZB cells than B6 MZB cells, or to FOB cells from either strain (Figure 7A). Moreover, B6.TC CD4**^+ ^**T cells proliferated more in response to B6.TC MZB cells than B6 MZB cells. Both MZB and FOB cells from B6.TC origin resulted in a greater CD4**^+ ^**T cell size than B6 B cells (Figure 7B). The greater T and B cell proliferation corresponded to a reduction of the percentage of CD4^+ ^T cells in the co-cultures (Figure 7C), which could correspond to either activation-induced cell death or to an outgrowth of the B cells. Regarding T cell activation, only B6.TC MZB cells induced a significantly increased CD69 expression (Figure 7D). Moreover, MZB cells significantly decreased CD62L expression on B6 CD4^+ ^T cells, and this decrease was enhanced with MZB cells from B6.TC origin (Figure 7E). CD62L expression on B6.TC CD4^+ ^T cells was not changed by co-culture with B cells, except with B6 FOB, which surprisingly increased CD62L (Figure 7E). Overall, these results showed that B6.TC MZB cells are more potent activators of CD4^+ ^T cells than B6 MZB cells, and that different patterns of activation may result from the intrinsic level of activation of the T cells themselves.

## Discussion

MZB cells constitute potent effector cells that can readily differentiate into plasma cells [8] and activate naïve CD4^+ ^T cells [4]. Moreover, MZB cells are enriched in autoreactive specificities and an enlargement of the MZB compartment has been implicated in murine lupus [15-17,29] and type 1 diabetes [30,31]. These results suggested that a controlled size of the MZB cell compartment and its sequestration within the MZ are important to maintain immune tolerance. Our own results in the NZM2410 model supported this hypothesis by showing an inverse correlation between the amount of B cells physically present in the MZ and autoimmune pathology [21,22]. In this study, we report that the breach of MZB cell follicular exclusion occurs early in B6.TC spleens, indicating that it is not a consequence of the progressive disorganization of the follicles associated with the lymphoid expansion and plasma cell accumulation in this strain [32]. It also indicates that the intrafollicular MZB cells have the opportunity to contribute to the early events in the disease process. We also demonstrated the existence of direct contacts between B6.TC MZB cells and CD4^+ ^T cells inside the follicles, through which these two cell types have the opportunity to activate each other.

The two models of autoreactive HC Tg B cells that we used, 56R and AM14, are directed against prototypic lupus antigens, DNA [23] and IgG2a^a^-chromatin ICs [33]. As previously reported for 56R B cells [23], the AM14 HC Tg B cells were preferentially recruited to the MZB compartment, reflecting their enrichment for autoimmune specificities. More importantly, both 56R and AM14 MZB cells were in significantly greater numbers inside the follicles than WT MZB cells. The expression of *Sle2 *locus alone for 56R, and more strongly of *Sle1-3 *for AM14 increased the shift of 56R and AM14 MZB cells towards the follicles. These results are in full support of our hypothesis that autoreactive MZB cells have a greater propensity for intrafollicular location, based on the specificity of their BCR and the expression of *Sle *susceptibility loci. The greater migratory behavior of B6.TC MZB cells either spontaneously (this study) or in response to CXCL13 or S1P [21] may contribute at least in part to their increased intrafollicular location. Further studies will be necessary to established whether these cells become trapped in the follicule.

Our *in vitro *experiments have confirmed established results: First, B6.TC B cells have greater effector functions than B6 B cells [20,34], and second, MZB cells respond more than FOB cells to TLR ligands [35,36] or to T cells [7,10], and are better activators of CD4^+ ^T cells [4]. Most importantly, our experiments demonstrated that the greater responsiveness of MZB cells is enhanced in lupus-prone B6.TC mice, making the B6.TC MZB cells a very potent effector population. In addition to TLR ligands, B6.TC MZB cells responded strongly to IC stimulation. The defective NZW allele of FcγRIIb [37] may contribute to this phenotype, although down-regulation of FcγRIIb^NZW ^on MZB cells has not been reported, in general and in the B6.TC strain (Morel, unpublished). The strong response of B6.TC MZB cells to chromatin ICs strongly suggest that these cells are enriched for anti-chromatin autoreactive specificities. Loss of tolerance to chromatin represents a critical step in the initiation of systemic autoimmunity in lupus [38,39], and it is likely that B6.TC MZB cells contribute to the autoimmune process though their reactivity to chromatin. Our results also showed a mutual activation between B6.TC MZB cells on B6 CD4^+ ^T cells, indicating that these MZB cells have the potential to activate non-autoreactive T cells. The strongest responses were however observed between B6.TC MZB and B6.TC CD4^+ ^T cells, which are likely to share autoreactive specificities, such as chromatin [28]. This suggests an amplification mechanism between B6.TC MZB and CD4^+ ^T cells in which MZB cells respond aberrantly to TLR or IC stimulation, secrete autoAb and move inside the follicles where they encounter CD4^+ ^T cells. Upon these encounters, the CD4^+ ^T cells become activated themselves and can in turn provide further activation signals to MZB cells or other cell types to participate in the autoimmune process.

## Conclusions

This study showed that the NZM2410 susceptibility loci induce MZB cells to locate into the follicles, and that this breach of follicular exclusion occurs early in the development of the autoimmune pathogenesis. The enhanced responses to stimulation and increased effector functions of MZB cells from lupus-prone mice as compare to non-autoimmune MZB cells provide a mechanism by which the failure of MZB cell follicular exclusion contributes to the autoimmune process. Overall, our results suggest a novel tolerance checkpoint in regulating systemic tolerance consisting of containing hyperactive MZB cells in the marginal zone away from T cells.

## Methods

### Mice

The triple congenic B6.NZM2410.*Sle.Sle2.Sle3 *(BcN/LmoJ in JAX^® ^Mice, referred to as B6.TC) [20] and C57BL/6J (B6) mice were maintained at University of Florida. B6.*Sle2*.56R and B6.56R breeders were a generous gift from Chandra Mohan (UTSW). B6.TC.AM14.IgH^a/b ^and B6.AM14.IgH^a/b ^were obtained by breeding B6.TC and B6 to B6.AM14.*lpr*, which carry the AM14 heavy chain (HC) transgene (Tg) (kindly provided by Mark Shlomchik, Yale Medical School) and C57BL/6J-*Cg-IghaThy1aGpila/J (*B6^a^) mice. The presence of the IgH^a ^allele was screened by flow cytometry with allotype-specific anti-IgM^a ^(Igh6a). The presence of the AM14 HC Tg was detected by PCR as previously described [24]. All *in vitro *experiments used 2 to 3 mo old females before any manifestation of clinical disease. All animal protocols were approved by the Institutional Animal Care and Use Committee of the University of Florida.

### Histology

Immunofluorescence staining was performed on frozen sections as previously described [22], with Moma-1-FITC (Serotec) or CD4-FITC (GK1.5), CD1d-PE (1B1), and IgM^a^-Biotin-SA-PB or B220-PB (RA3-6B2). Quantitation was performed with a MetaMorph 7.5 image analysis station, which quantifies the number of pixels for a given color corresponding to a specific stain combination. For each B6.TC and B6 mice, the numbers of CD1d^+ ^B220^+ ^cells and CD1d^- ^B220^+ ^were computed in four areas with homogeneous staining selected on each side of the Moma-1^+ ^ring on 100X or 200X amplification images. Five mice were evaluated for each strain and age group.

### Cell cultures

B220^+ ^CD23^- ^CD21^hi ^MZB and B220^+ ^CD23^+ ^CD21^int ^FOB cells were purified on a FACSAria cell sorter, yielding 95-98% purity in both strains. We used the anti-CD21 7E9 Ab which binds equally the B6 and NZW alleles of CD21 [40]. CD4^+ ^T cells were enriched by negative selection with magnetic beads (Myltenei). CFSE-labeled MZB or FOB cells (10^5 ^per well) were cultured with or without CFSE-labeled CD4^+ ^T cells in RPMI 1640 containing 10% FCS for either 3 or 5 d with LPS (Sigma, 5 ug/ml), ODN1826 CpG (InvivoGen, 1 ug/ml), anti-CD40 (R&D Systems, 10 ug/ml), anti-chromatin or protein ICs (50 ug/ml final Ab concentration). ICs were prepared as previously described [41]. Briefly, chromatin-enriched 0.2 μM filtered supernatant (12.5% of total well volume) from B6.TC spleen cells cultured for 48 h, or TNP-BSA (12.5 ug/ml, Biosearch Technologies) were incubated with the PL2-3 IgG2a anti-chromatin Ab or with anti-TNP IgG2a (BD pharmingen), respectively, for 30 min before the assay. In some cultures, CD4^+ ^T cells (1×10^6^/ml) were pre-activated overnight with 1 μg/ml of PMA and 1 μM ionomycin. In each assay, 2-3 mice per strain were used and each result reported in this study is representative of at least 2 independent assays. Culture supernatants were assayed at 1:10 dilution for total IgM, anti-ssDNA and anti-dsDNA IgM by ELISA carried out as previously described [42]. Spontaneous migration of MZB and FOB cells was measured intranswell assays as previously described [21] with an initial input of 2 × 10^6 ^negatively selected total splenic B cells.

### Flow cytometry

The following conjugated mAbs were used: CD23 (B3B4), CD1d (1B1), CD21 (7E9), CD69 (H1.2 F3), CD62L (MEL14), CD138 (281-2), B220 (RA3-6B2), IgM (II/41), CXCR5 (2G8), all, except 7E9, purchased from BD Pharmingen. Stained cells were analyzed on FACSCalibur cytometer (BD Biosciences). Dead cells were excluded based on forward and side scatters characteristics.

### Statistical analysis

Data were analyzed with the GraphPad Prism 4.0 software package with the appropriate tests as indicated in the text.

## Abbreviations

SLE: systemic lupus erythematosus; MZ: marginal zone; BCR: B cell receptor; FO: follicular; B6.TC: B6.NZM2410.*Sle.Sle2.Sle3 *or BcN/LmoJ; Ab: antibody; HC: immunoglobulin heavy chain; Tg: transgenic; IC: immune complex; B6: C57BL/6J; SEM: standard error of the mean.

## Competing interests

The authors declare that they have no competing interests.

## Authors' contributions

ZZ participated in the study design, performed the *in vitro *experiments and the morphometric analysis; HN performed the immunofluorescence histology and assisted with the *in vitro *experiments; YYZ performed the migration assays, LM conceived the study, performed the statistical analysis and wrote the manuscript. All authors read and approved the final manuscript.
